# Metallic Graphene Nanoribbons

**DOI:** 10.1007/s40820-020-00556-5

**Published:** 2021-01-05

**Authors:** Sheng-Yi Xie, Xian-Bin Li

**Affiliations:** 1 School of Physics and Electronics, Hunan University, 410082 Changsha, People’s Republic of China; 2 State Key Laboratory of Integrated Optoelectronics, College of Electronic Science and Engineering, Jilin University, 130012 Changchun, People’s Republic of China

**Keywords:** Graphene, Nanoribbons, Quantum confinement effect, Supperlattice

## Abstract

Isolated graphene nanoribbons (GNRs) usually have energy gaps, which scale with their widths, owing to the lateral quantum confinement effect of GNRs. The absence of metallic GNRs limits their applications in device interconnects or being one-dimensional physics platform to research amazing properties based on metallicity. A recent study published in *Science* provided a novel method to produce metallic GNRs by inserting a symmetric superlattice into other semiconductive GNRs. This finding will broader the applications of GNRs both in nanoelectronics and fundamental science.

Unlike the gapless semimetal of graphene [1], the graphene nanoribbons (GNRs) [2], whether armchair or zigzag type, usually own an energy gap scaling inversely with their widths due to the lateral quantum confinement effect of GNRs [3]. The raised energy gap, which is absent in graphene, enables the production of transistor [4], yet the robust semicondutivity of GNRs limits the applications in such as device interconnects or being one-dimensional physics platform to explore superconductivity [5], Luttinger liquid [6], charge density waves [7] or spintronics [8]. Recently, one paper published in *Science* reported an ingenious method to produce metallic GNRs [9] based on the atomically precise bottom-up synthesis.

In this work, Rizzo et al. in University of California at Berkeley used the precursor molecule 1 (Fig. 1a) to construct the GNRs with the symmetrical insertion of methyl groups to form the superlattice, which are named as the sawtooth GNR (sGNR). Upon annealing over 350 ℃, the sGNRs transformed to the called five sawtooth sGNRs (5-sGNRs) with minor chemical bond rearrangements to form a five-membered ring along their edges, as also shown in Fig. 1a. Electronic structures of sGNRs and 5-sGNRs were further determined both by scanning tunneling microscope (STM) spectroscopy and density functional theory (DFT) calculations. The experimental d*I*/d*V* point spectrum of a sGNR is shown in Fig. 1b, and the sharp peak states as well as their projection in real space (Fig. 1c) at the zero bias clearly show the metallic density of states (DOS), which agrees well with the DFT results (Fig. 1d, e). In their further experiment, the DOS of 5-sGNRs spans a broader energy range around the Fermi level, inducing the robust metallicity with a 20-fold increase of the metallic bandwidth, as shown in Fig. 1.

**Fig. 1:**
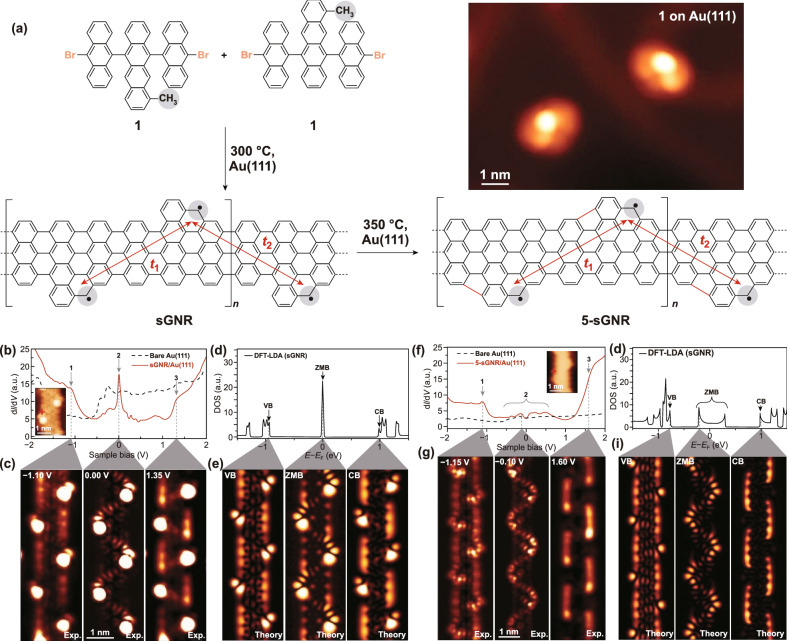
**a** Schematic bottom-up growth of sGNRs and 5-sCNRs from molecular precursor 1 on Au (111), and the inset at the top right corner shows STM topograph of two isolated monomers of precursor 1. **b-e** Electronic structure of sGNRs. **b** experimental d*I*/d*V* point spectroscopy, **c** related constant-height d*I*/d*V* maps in real space, **d** DFT-LDA calculated DOS, and **e** related local DOS maps of a sGNR. **f-i** Electronic structure of 5-sCNRs. **f** experimental d*I*/d*V* point spectroscopy, **g** related constant-height d*I*/d*V* maps, **h** DFT-LDA calculated DOS, and **i** related local DOS maps of a 5-sCNR. Reproduced from Ref. [9]. Copyright 2020 American Association for the Advancement of Science.

This work provides a smart strategy for realizing the metallicity in GNRs to act as a candidate used in logic devices. In future, as a direct measurement of metallicity, variable temperature conductivity experiments can be further considered. Additionally, the performance comparison between these metallic GNRs and traditional metals like copper used in interconnect technology is also meaningful for the applications in nanoelectronics. Finally, considering the possible formation of junctions between these metallic GNRs and ordinary semiconductive GNRs, whether Ohmic contact or Schottky contact can be formed should also deserve extra efforts.
